# Medical Image Compression Based on Vector Quantization with Variable Block Sizes in Wavelet Domain

**DOI:** 10.1155/2012/541890

**Published:** 2012-09-19

**Authors:** Huiyan Jiang, Zhiyuan Ma, Yang Hu, Benqiang Yang, Libo Zhang

**Affiliations:** ^1^Software College, Northeastern University, Shenyang 110819, China; ^2^Key Laboratory of Medical Image Computing, Ministry of Education, Shenyang 110819, China; ^3^Department of Radiology, Chinese PLA General Hospital, Shenyang 110015, China

## Abstract

An optimized medical image compression algorithm based on wavelet transform and improved vector quantization is introduced. The goal of the proposed method is to maintain the diagnostic-related information of the medical image at a high compression ratio. Wavelet transformation was first applied to the image. For the lowest-frequency subband of wavelet coefficients, a lossless compression method was exploited; for each of the high-frequency subbands, an optimized vector quantization with variable block size was implemented. In the novel vector quantization method, local fractal dimension (LFD) was used to analyze the local complexity of each wavelet coefficients, subband. Then an optimal quadtree method was employed to partition each wavelet coefficients, subband into several sizes of subblocks. After that, a modified *K*-means approach which is based on energy function was used in the codebook training phase. At last, vector quantization coding was implemented in different types of sub-blocks. In order to verify the effectiveness of the proposed algorithm, JPEG, JPEG2000, and fractal coding approach were chosen as contrast algorithms. Experimental results show that the proposed method can improve the compression performance and can achieve a balance between the compression ratio and the image visual quality.

## 1. Introduction

With the rapid development of modern medical industry, medical images play an important role in accurate diagnosis by physicians. However, the large amount of images put forward a high demand on the capacity of the storage devices. Besides, telemedicine is a development trend of medical industry, while narrow transmission bandwidth limits the development of this project. To solve the problems mentioned above, a large number of researches have been carried out into medical image compression.

Medical image compression approaches can simply be divided into two kinds: lossless compression and lossy compression. Lossless compression can reconstruct the original image completely identical. Lossy compression takes advantage of the human weak psychovisual effects to optimize compression results but loses certain image information [1]. Lossless coding method like them Huffman coding [2], LZW [3], arithmetic coding [4], and some other improved methods [5] can code an image and decode it with perfect result. However, such methods can only obtain a low compression ratio around 1 to 4; a higher compression ratio is hard to obtain. Although physicians and scientists prefer to work with uncorrupted data, the modest compression offered by lossless coding is often insufficient for either transmission or storage purpose. In these cases, a lossy compression method that preserves the diagnostic information is needed. There are lots of lossy image-coding methods, such as predictive coding [6], discrete cosine transformation (DCT) coding [7], fractal-based coding [8], wavelet-based coding [9, 10], vector quantization (VQ) method [11, 12], and some other methods. Predictive coding approach can mostly reduce the relevance of pixels in time and space domain. This kind of method can get a good visual quality for decoded image, but its compression ratio is just ordinary. Wavelet-based methods are commonly used in image coding, which can analyze an image in different resolutions. DCT is easily implemented and widely used in image coding, and it can decode an image within short time, but the compression ratio is limited, or it may bring block effect to its decoded image. The fractal image coding possesses excellent visual quality and compression ratio. However, it has some limitations like exhaustive inherent coding time. There are some modified methods aiming at shortening the coding time of the fractal method, which can be found in [13]. Artificial neural network (ANN) [14] method is a late-model image-coding method and is widely used, but the training of its samples is time consuming and the choice of the neural network model is vital in image coding. There are many kinds of image-coding algorithms relating to wavelet transform, like embedded zero-tree wavelet (EZW) [15], set partitioning in hierarchical trees (SPIHT) [16], and some other methods. Those methods have achieved great success in recent years. However, all of these methods could not provide a high compression ratio.

VQ is an efficient information source coding method. The principle of it is constructing a vector based on several scalar data group, so the vector quantization coding is superior to scalar quantization coding. LBG algorithm was named after its proposers Linde et al. and afterwards there were many other improved methods [17]. This method can provide a high compression ratio and a simple decoding process. However, a serious problem in traditional VQ is edge degradation. To solve the problem, variable block-size image coding was introduced by Vaisey and Gersho [18]: the technique obtained a satisfactory quality coding result at a high ratios. Variable block-size image coding is based on the traditional vector quantization. Rather than splitting the image into series of sub-blocks in uniform size like the traditional vector quantization algorithm, variable block-size coding segments the original image into several types of blocks. In the process, areas which present high complexity are divided into small blocks, while those of low complexity are divided into large blocks. The variable block coding method can achieve a high visual quality and a relatively high compression ratio. Related works on the vector quantization of variable block have been reported by Yamanaka [19], which performs the variable block coding in the wavelet domain. Besides, Sasazaki et al. [20] used local fractal dimension as a metric to evaluate the complexity of a local area of image.

This paper introduces a novel approach for medical image compression based on wavelet transformation and vector quantization, which could provide an efficient compression performance with good visual quality. In our scheme, as most energy of the original image concentrates in the lowest-frequency subband, we decompose the original image into one low frequency subimage and several high-frequency subimages using wavelet transformation The low-frequency subimage is manipulated with the Huffman coding as its information is very sensitive to human's eyes. Information in high-pass octave bands is always the details or edges. According to the human visual system (HVS), the loss of this part will not be perceived. So, we process the high-frequency subimages with our proposed vector quantization approach.

## 2. Outline of Our Proposed Scheme

**Figure 1:**
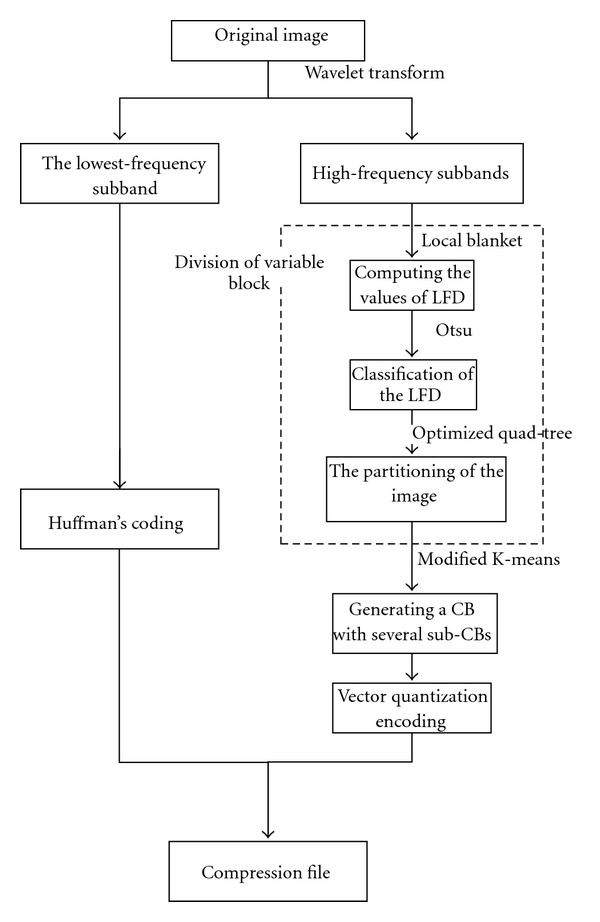
The diagram of the proposed algorithm.

In our scheme, an original image is first decomposed into multiresolution subimages through the wavelet transform; then, coefficients in the low-pass octave bands are encoded by the Huffman coding method while coefficients in each of the high-pass octave bands are encoded by the proposed novel vector quantization with variable block. The diagram of the proposed algorithm is shown in Figure 1.

The Huffman coding method is a common method in the field of image compression, so the introduction of this part is ignored. The optimized vector quantization with variable block size includes two steps. The first step is dividing the subimage into series of sub-blocks. The smooth areas of the coefficient matrix are divided into sub-blocks with a relatively large size, while those containing many details are divided into small-size sub-blocks. The metric of complexity is based on the value of local fractal dimension (LFD). In order to classify the local fractal dimension into predetermined classes, a method of discriminant analysis is applied [21]. After that, an optimized quadtree (QT) approach is exploited to split the subimage into different sizes of blocks. The second step is quantization step. In the procedure, every codebook is made up of several sub-books. Each of the sub-books is trained by a modified *K*-means method. Then each of the subimages is encoded and decoded by the obtained codebook.

## 3. Medical Image Compression Based on Our Proposed Algorithm

### 3.1. Dividing High-Frequency Sub-Bands Into Variable-Size Blocks

Fractal dimension (FD) is a statistical quantity that gives an indication of how completely a fractal object appears to fill space. The value of FD has a strong relevance with the human judgment of surface roughness. In this sense, FD is an excellent tool in the field of image processing. The fractal dimension measurement cannot be derived exactly but must be estimated. In this paper, the blanket method [22] is used to estimate the local fractal dimension of images. In the technique, the subimage composed by high-pass coefficients can be viewed as a hilly terrain surface whose height from the normal ground is proportional to the value of the coefficients [23]. Then all points at distance from the surface on both sides generate a blanket of thickness 2*ε*. The covering blanket is defined by its upper surface *u*_*ε*_ and *b*_*ε*_ [24]. The surface area of the blanket is calculated repeatedly. The initial surface of the blanket, *u*_0_ and *b*_0,_ is set by the values of coefficients of the subimage. Then, the surfaces of different *ε* are defined by

(1) uε(i,j)=max⁡{uε−1(i,j)+1,max⁡d(i,j,m,n)≤1⁡uε−1(m,n)},bε(i,j)=min⁡{bε−1(i,j)−1,min⁡(m,n)−(i,j)≤1⁡bε−1(m,n)},

where *d*(*i*, *j*, *m*, *n*) is the distance between the pixel (*i*, *j*) and pixel (*m*, *n*). In this formula, the points (*m*,*n*) are taken to be the four immediate neighbors of the point (*i*, *j*). The volume of the blanket is calculated from

(2) vε=∑i,j(uε(i,j)−bε(i,j)),

while the surface area is measured as

(3) A(ε)=(vε−vε−1)2.

On the other side, the area of a fractal surface behaves according to

(4) A(ε)=fε2-D,

where *F* is a constant for a specific image and *D* denotes the fractal dimension of an object.

Therefore, the *FD* value can be estimated from the linear fit of log⁡⁡{*A*(*ε*)} against log⁡⁡{*ε*} with the blanket's scale range from 1 to *ε*, and the slope should be equal to 2D. As to the estimation of local fractal dimension, the window size of the local area is always 3 × 3, 5 × 5, 7 × 7, or larger.

After the local complexity analysis of the subimage, a LFD mapping image is obtained. In the LFD mapping image, the pixel value of each point is the local fractal dimension of the corresponding pixel in the original subimage. According to the local fractal dimension, the image is divided into sub-blocks of different sizes. Because all the LFD values of the subimage are being decimal in the range of [2, 3], it is impossible to split the subimage into so many types of blocks as the number of local fractal dimension shows. So we must classify the LFD mapping image into a certain number of classes. In the procedure, discriminant analysis [21] is applied. This technique can select a threshold adaptively, which will be used to divide an image into object and background.

In this paper, an optimized quadtree method is performed to decompose the subimage into several sizes of blocks. In order to understand the optimized quadtree method, the quadtree algorithm is first explained as follows.

Quadtree is a tree data structure which was named by Finkel and Bentley in 1974 [25]. In quadtree decomposition, a judgment is first made to see whether a block can be represented by a single large block or it must be divided into four sub-blocks [26]. In this paper, the classification result of local fractal dimension is seen as the judgment in the process of quadtree decomposition. The quadtree technique recursively subdivide the image into four quadrants or regions until the priority of the current block is homogenous.

During the process of variable block division, what sizes of block a pixel will be contained is not only depending on the priority of itself, but also on the priority of its surrounding pixels. So we can draw a conclusion that adjacency relationship is very important to each pixel. As for the whole sub-band, if we can find a good adjacency relationship for all pixels, the proportion of different sizes of image blocks will be changed and the quality of decoded subimage will also be changed. The purpose of the optimized quadtree is to select an excellent initial position to split the subimage, which will improve the compression ratio or boost the image quality at the same bit ratio.

**Figure 2:**
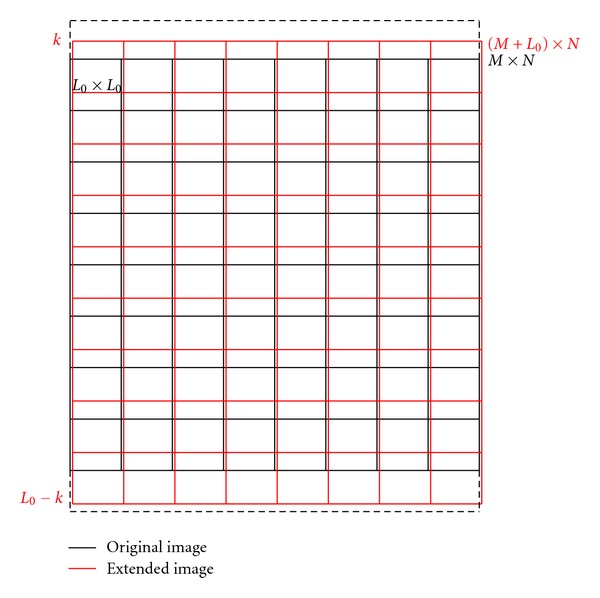
Schematic diagram of the optimized quadtree method.

In conventional quadtree method, the image is first divided into many initial sizes of sub-blocks from the first row to the last, from the first column to the last column, then the initial sub-blocks will continue to split. In the proposed technique, the initial split position will change regularly and then the best initial split position is chosen to process the initial division of the image. The novel method consists of two steps: statistics step and selection step. The schematic diagram of the optimized quadtree method is shown in Figure 2.

Assume that the size of the initial sub-block is denoted as *L*_0_; the size of original subimage is *M* × *N*, where *M* is number of rows and *N* is number of columns of the subimage. In the proposed method, there are two ways to change the position. The first way is fixing the left side of the initial segment position at the first column, while changing the upper side from 1 to *L*_0_ line. The second way is changing the left side of the initial segment position from 1 to *L*_0_ − 1 column, while fixing the upper side at the first line. In this way, there are 2*L*_0_ − 1 types of positions. To ensure the integrity of the original subimage, the subimage will be extended in a certain way when changing the position. For instance, if the initial position is changing at the row direction and the original line of the split location is the *k*th line, the upper part of the image will be extended by *k* rows and the lower part of the image will be extended by *L*_0_ − *k* rows. A similar process will be performed when the changing position is towards the column direction. The extended part is simply a copy of the first line or the last line. In this way, the size of the original subimage is expanded, so at the decode side, the decoded subimage should be shrunk in a corresponding way.

At each of the initial positions, variable block division is performed. And in this process, the numbers of different sizes of image blocks will be counted and the structural similarity (SSIM) between the division image and the LFD mapping image will be calculated. After that, the best initial position, which will lead to the largest summation of the structure similarity (SSIM) and the number of the smallest sub-blocks, will be chosen as the final initial split position to segment the subimage. The structure similarity will be introduced in detail in the following part. Structure similarity is a numerical in the interval of [0, 1], while the number of the highest-priority sub-blocks is approximate to 10^5^. So the structure similarity should be multiplied by 10^5^ before the operation mentioned above. The way to choose the best initial split position is defined by

(5) SUMi=Ni+105SSIMi, i=1,2,…,2L0−1,SUMmax⁡=max⁡{SUMi}, i=1,2,…,2L0−1.

Here, SUM_*i*_ is summation of the structure similarity and number of the smallest-size sub-blocks. *N*_*i*_ is the number of the highest-priority sub-blocks in the *i*th initial split position.

The SSIM [27] is an objective image quality metric, which plays a variety of roles in image processing. It can be used to adjust image quality; in addition, it can be used to optimize algorithms and parameters settings of an image processing system. It is exploited to optimize algorithm in this paper.

The SSIM separates the task of similarity measurement into three comparisons: luminance, contrast and structure. The SSIM between two images is defined by

(6) SSIM(x,y)=[l(x,y)]α[c(x,y)]β[s(x,y)]γ,

where *α*>0,  *β*>0, and *γ*>0 are parameters used to adjust the relative importance of the three components. The formula *l*(*x*, *y*) indicates the luminance comparison of the two images. *c*(*x*, *y*) indicates the contrast comparison of the two images. *s*(*x*, *y*) indicates the structure comparison of the two images.

In order to simplify the expression, we set *α* = *β* = *γ* = 1, which means that the luminance, contrast and structure comparisons of two different images will plays the same important role on the similarity measurement. The result of the specific form of SSIM is defined as follows:

(7) SSIM(x,y)=(2uxuy+C1)(2σxy+C2)(ux2+(uy2)+C1)(ux2+uy2+C2),

where *u*_*x*_, *u*_*y*_ is the mean intensity of the image *x* and *y*: respectively, *σ*_*xy*_ is the covariance between the image *x* and *y*; *C*_1_ and *C*_2_ are constants, which are used to avoid instability when *u*_*x*_^2^ + *u*_*y*_^2^ is very close to zero.

### 3.2. Generating a Codebook with Several Subcodebooks

*K*-means algorithm is an useful cluster algorithm, which divides *N* data points in an *I*-dimensional space into *K* clusters. Each cluster is parameterized by a vector *m* ^*k*^, which indicates the center of each cluster. To start the *K*-means algorithm, the sets of {*m* ^*k*^} are initialized in some way. Then, the algorithm will come into an iterative two-step phase: assignment and update step. In the assignment step, each data point *n* is assigned to the nearest mean, and in the update step, the means are adjusted to match the sample means of the data points. When the algorithm reaches the predetermined maximum iterating time or the incremental error is below the predetermined threshold, the iteration is over.

One disadvantage of the *K*-means method is that it is very easy to fall into a local optimal result. The performance of it depends on the initial clusters seriously. So, a good initial codebook can efficiently reduce the iteration times in the process of training codebook and greatly improve the quality of the decoded image.

A common used method to initialize codebook is the Forgy and Random Partition [28]. It randomly chooses *k* observations from the data set and uses these data as the initial means while the Random Partition method first randomly assigns a cluster to each observation and then carries out an update step, thus computing the initial means to be the centroid of the cluster's randomly assigned points. The Forgy method tends to spread the initial means out, while Random Partition places all of them close to the center of the data set. According to Hamerly and Elkan [29], the Random Partition method is generally preferable.

In this paper, we introduce a new method to set initial code vectors of the codebook, which is based on the energy of each sub-block. In the proposed method, we first calculate the energy of each sub-block. The energy function is defined as

(8) ∑i=1Lkxi2,

where *L*_*k*_ is the number of pixels in the *k*th sub-block and *x*_*i*_ is the gray level of the pixel *i*. Then the energy of all of the sub-blocks is ordered, and the predetermined numbers of code vectors are taken out in equal interval. In the novel method, the image information is taken into account at the process of selecting the initial codebook.

## 4. Results and Discussions

To validate the performance of the proposed medical image compression algorithm, 30-pair liver CT digital imaging and communications in medicine (DICOM) images and brain CT DICOM images from a 64 row CT machine in a domestic large hospital were tested. The resolution of all the images is 512 × 512. In the process of wavelet transform, the biorthogonal wavelet basis of bior4.4 is chosen to transform the medical images. The results through different wavelet basis do not vary obviously. To ensure that most of the image energy is not lost, two-level wavelet decomposition is executed in the experiment.

After that, the LFD value of each high-frequency subband will be classified into three categories by the discriminant analysis approach, and then each sub-band is divided into 2 × 2, 4 × 4, and 8 × 8 sub-blocks. In order to prove the effectiveness of proposed codebook training method, we will construct two kinds of codebooks for every sub-band. One kind of codebook is generated by K-means which is based on the random initial codebook, while the other one is obtained from the energy function initial codebook. Therefore, there are six sub-codebooks for one subimage, which are generated from the 2 × 2, 4 × 4, and 8 × 8 sub-blocks of the image, respectively. In the subsequent part of the paper, the codebook constructed by the random initial codebook is called random codebook, and the codebook constructed by the energy function initial codebook is called energy codebook.

**Figure 3:**
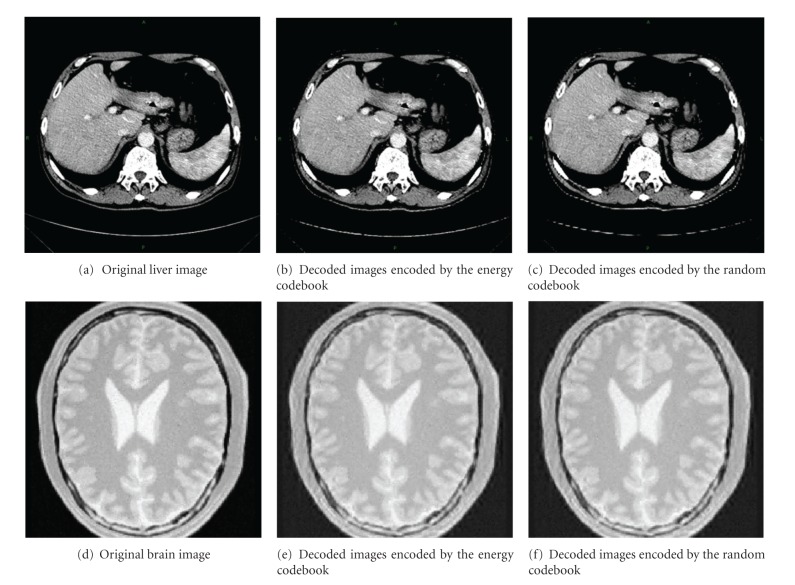
Comparison of the decoded images of the energy codebook and the random codebook at compression ratio around 10.

Liver CT images and brain CT images are used to test the proposed method.  Figure 3 shows the reconstructed images of the different codebooks. Figures 3(a) and 3(d) are original images, Figures 3(b) and 3(e) are decoded images encoded by the energy codebook, and Figures 3(c) and 3(f) are decoded images encoded by the random codebook. The calculation of compression ratio should take both of the lossless part and lossy part into account. The compression ratio of lossless part is 1.5097, and the lossy part is 15.4451, so the real compression ratio is 9.7943. As for the head image, the compression ratio of lossless part is 1.3458, and that of the lossy part is 15.3250, the real compression ratio is 9.5363.

**Figure 4:**
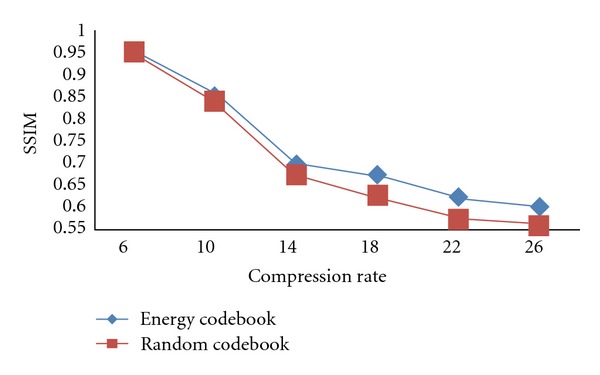
Average SSIM for liver images against compression ratio.

**Figure 5:**
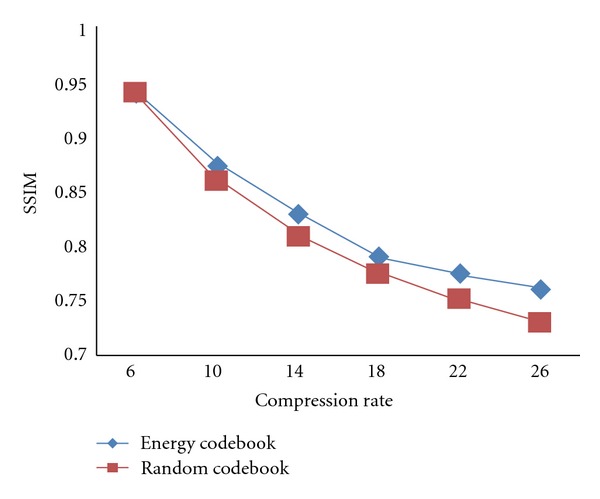
Average SSIM for head images against compression ratio.

As shown in Figure 3, we can see that the qualities of two images decoded with different codebooks are almost the same when the compression ratio is fixed. However, the contour of the abdominal of the energy codebook is clearer than the random codebook. Besides, the SSIM value of the decoded image of energy codebook and the original image is higher than that of random codebook by 0.02. As to the head image, the fidelity of the two decoded images is lower than that of liver images, but it will not cause diagnosis failure. The contrast of the reconstructed image of the energy codebook is superior to that of the random codebook, and the SSIM between the energy decoded image and the original image is higher than that of random decoded image. The average SSIM values of 30 liver images and 30 head images decoded by the two kinds of codebook at different compression ratio are illustrated in Figures 4 and 5.

As shown in Figures 4 and 5, at low compression ratio, the performance of the energy codebook is similar to that of the random codebook. However, the performance of the energy codebook is obviously superior to the random codebook at high compression ratio.

**Figure 6:**
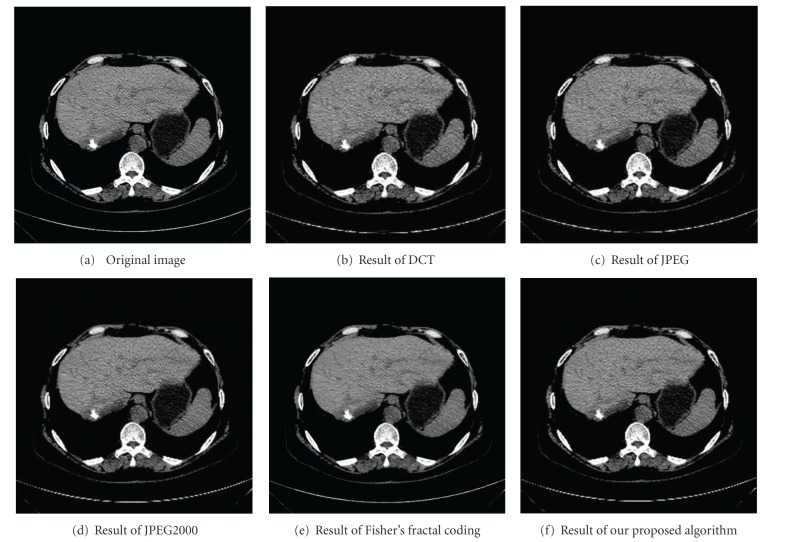
Contrast of compression results at a compression ratio of 25.

In order to verify the effectiveness of our proposed algorithm, we compare our algorithm with several recent and conventional compression methods, such as DCT, Fisher's fractal coding, JPEG, and JPEG2000. The corresponding contrast results are shown in Figure 6(a) for an original liver image; 6(b), 6(c), 6(d), 6(e), 6(f) are, respectively, the compression results of the DCT, JPEG, JPEG2000, Fisher's fractal coding and our proposed algorithm at a compression ratio of 25.

As shown in Figure 6, when the compression ratio is up to 25, liver image compressed by all the above algorithms exist distortion phenomenon. However, the texture feature of the de-compressed image with our proposed algorithms is better than those in images with other methods.

We take peak signal-to-noise ratio (PSNR), mean squared error (MSE), the running time, and normalized cross-correlation (NCC) as four evaluation indexes to compare our proposed algorithm with the contrast methods. When the compression ratio is fixed to 25, the value of the above evaluation index is shown in Table 1.

As shown in Table 1, the PSNR and NCC values of our proposed algorithm are higher than the other four contrast methods, while the running time and MSE values of it's lower than the other contrast methods.

## 5. Conclusions

In this paper, we propose an optimized medical image compression algorithm based on wavelet transform and vector quantization with variable block size. We, respectively, manipulate the low-frequency components and high-frequency components with the Huffman coding and improved VQ by the aids of wavelet transformation. Although the implement of the proposed algorithm is more complex than some traditional medical image compression algorithms, it can compress an image with good visual quality with high compression ratio. Besides, in contrast with some traditional and current medical image compression algorithm, the proposed algorithm owns better performance through the evaluation index of PSNR, running time, MSE, and NCC.

## Figures and Tables

**Table 1:** Evaluation index value of our proposed algorithm and some contrast algorithms with compression ratio of 25.

	PSNR (dB)	Time (s)	MSE	NCC
DCT	27.24	13.22	37.12	0.9945
JPEG	28.11	14.65	35.65	0.9950
JPEG2000	34.16	12.20	24.94	0.9988
Fisher's method	31.54	10.50	33.66	0.9968
The proposed method	37.5	2.34	11.70	0.9996
